# Analysis of Two Novel Midgut-Specific Promoters Driving Transgene Expression in *Anopheles stephensi* Mosquitoes

**DOI:** 10.1371/journal.pone.0016471

**Published:** 2011-02-04

**Authors:** Tony Nolan, Elisa Petris, Hans-Michael Müller, Ann Cronin, Flaminia Catteruccia, Andrea Crisanti

**Affiliations:** 1 Department of Life Sciences, Imperial College London, London, United Kingdom; 2 Heidelberg University Biochemistry Centre, Heidelberg University, Heidelberg, Germany; 3 University of Perugia, Perugia, Italy; Universidade Federal do Rio de Janeiro, Brazil

## Abstract

**Background:**

Tissue-specific promoters controlling the expression of transgenes in *Anopheles* mosquitoes represent a valuable tool both for studying the interaction between these malaria vectors and the *Plasmodium* parasites they transmit and for novel malaria control strategies based on developing *Plasmodium*-refractory mosquitoes by expressing anti-parasitic genes. With this aim we have studied the promoter regions of two genes from the most important malaria vector, *Anopheles gambiae*, whose expression is strongly induced upon blood feeding.

**Results:**

We analysed the *A. gambiae Antryp1* and *G12* genes, which we have shown to be midgut-specific and maximally expressed at 24 hours post-bloodmeal (PBM). *Antryp1*, required for bloodmeal digestion, encodes one member of a family of 7 trypsin genes. The *G12* gene, of unknown function, was previously identified in our laboratory in a screen for genes induced in response to a bloodmeal. We fused 1.1 kb of the upstream regions containing the putative promoter of these genes to reporter genes and transformed these into the Indian malaria vector *A. stephensi* to see if we could recapitulate the expression pattern of the endogenous genes. Both the *Antryp1* and *G12* upstream regions were able to drive female-predominant, midgut-specific expression in transgenic mosquitoes. Expression of the *Antryp1*-driven reporter in transgenic *A. stephensi* lines was low, undetectable by northern blot analysis, and failed to fully match the induction kinetics of the endogenous *Antryp1* gene in *A. gambiae*. This incomplete conservation of expression suggests either subtle differences in the transcriptional machinery between *A. stephensi* and *A. gambiae* or that the upstream region chosen lacked all the control elements. In contrast, the G12 upstream region was able to faithfully reproduce the expression profile of the endogenous *A. gambiae* gene, showing female midgut specificity in the adult mosquito and massive induction PBM, peaking at 24 hours.

**Conclusions:**

Our studies on two putative blood-meal induced, midgut-specific promoters validate the use of *G12* upstream regulatory regions to drive targeted transgene expression coinciding spatially and temporally with pre-sporogonic stages of *Plasmodium* parasites in the mosquito, offering the possibility of manipulating vector competence or performing functional studies on vector-parasite interactions.

## Introduction

Anopheline mosquitoes are the most efficient vectors of the *Plasmodium* species that cause human malaria, a disease responsible for more than one million deaths per year[1]. Germline transformation technology for both *A. gambiae* and *A. stephensi* (the major malaria vectors in Africa and India respectively) now exists, opening the possibility to understand mechanisms of vector competence and the prospect of engineering *Plasmodium*-refractory anopheline mosquitoes. Irrespective of the molecular mechanism designed to attack the *Plasmodium* parasite, the development of malaria-refractory mosquitoes ultimately relies on the time- and tissue-regulated expression of anti-parasitic genes in concomitance with the appearance of target parasite developmental stages in order to maximise the effectiveness of transgene expression while minimizing the potential for reduced vigor associated with indiscriminate expression. This goal has driven the successful search for several tissue-specific promoters capable of driving expression in mosquito tissues relevant to parasite transmission, including the midgut, hemolymph, fat body and salivary glands[2], [3], [4], [5], [6]. In particular, blood meal-induced gut-transcribed promoters are ideal candidates to drive the expression of exogenous genes to attack the malaria parasite while it completes its life cycle within the mosquito host. This notion originates from the observation that the pre-sporogonic forms of the malaria parasite that develop within the midgut, from the gametocyte to the ookinetes, represent a major bottleneck in the parasite life cycle[7]. These forms, whose development is complete within 24 hours after a blood meal, expose on their surface a series of antigens that could be targeted by antibodies or other effector molecules before traversal of midgut epithelium is achieved[8]. Indeed, in the two laboratory-selected refractory mosquito strains, malaria parasite development is in fact blocked in the gut, either at the ookinete or the early oocyst developmental stages[9], [10]. Moreover, successful attempts in the past to engineer *Plasmodium*-refractory mosquitoes have all employed midgut-specific promoters to drive anti-parasitic gene expression[2], [11], [12], [13], [14].

Several strategies have been proposed to create refractory mosquitoes, including silencing the expression of essential molecules through RNAi in host cells invaded by the parasite, expression of toxins or single chain antibodies in close proximity to the invading parasite, and production of peptides to out-compete parasite binding to host epithelial receptors[2], [12], [13], [14], [15], [16]. Given the diversity of *Plasmodium* stages present within the midgut and the range of possible effector molecules, it is desirable to have a wide-range of promoters to target transgene expression most effectively to each particular stage.

In *A. gambiae*, a tightly clustered family of 7 gut-specific trypsin genes involved in digestion processes has been extensively studied[17], [18]. These genes are differentially regulated and the two late trypsins, *Antryp1* (AGAP008296) and *Antryp2* (AGAP008295), are induced upon a blood meal. In particular, *Antryp1* is highly expressed in female guts and its induction peaks at 24 hours after blood feeding[17]. The region immediately upstream of *Antryp1* has previously been shown as sufficient to direct midgut-specific expression in transgenic *Drosophila melanogaster* [19], a species evolutionally divergent from *Anopheles* by 250 million years[20].However, in that model the female-predominant expression was not maintained, and the *Antryp1* promoter region did not respond to a “blood meal-related” induction process.

The *G12* gene (AGAP006718), of unknown function, is similar to a number of cockroach allergens, and was initially identified in our laboratory in a screen for cDNAs that were enriched in *A. gambiae* after a bloodmeal. Our detailed analysis of the expression of *G12* shows it to be expressed exclusively in the female midgut in response to a bloodmeal.

Here we describe and compare the temporally and spatially directed expression in *A. stephensi* mosquitoes of reporter genes driven by the putative promoter regions of the *A. gambiae Antryp1* (pAntryp1) and *G12* (pG12) genes. Both promoters could be of potential use as candidates to drive the time- and tissue-specific expression of transgenes in *Anopheles* mosquitoes.

## Results and Discussion

### Validation of pAntryp1 in transgenic mosquitoes

**Figure 1:**
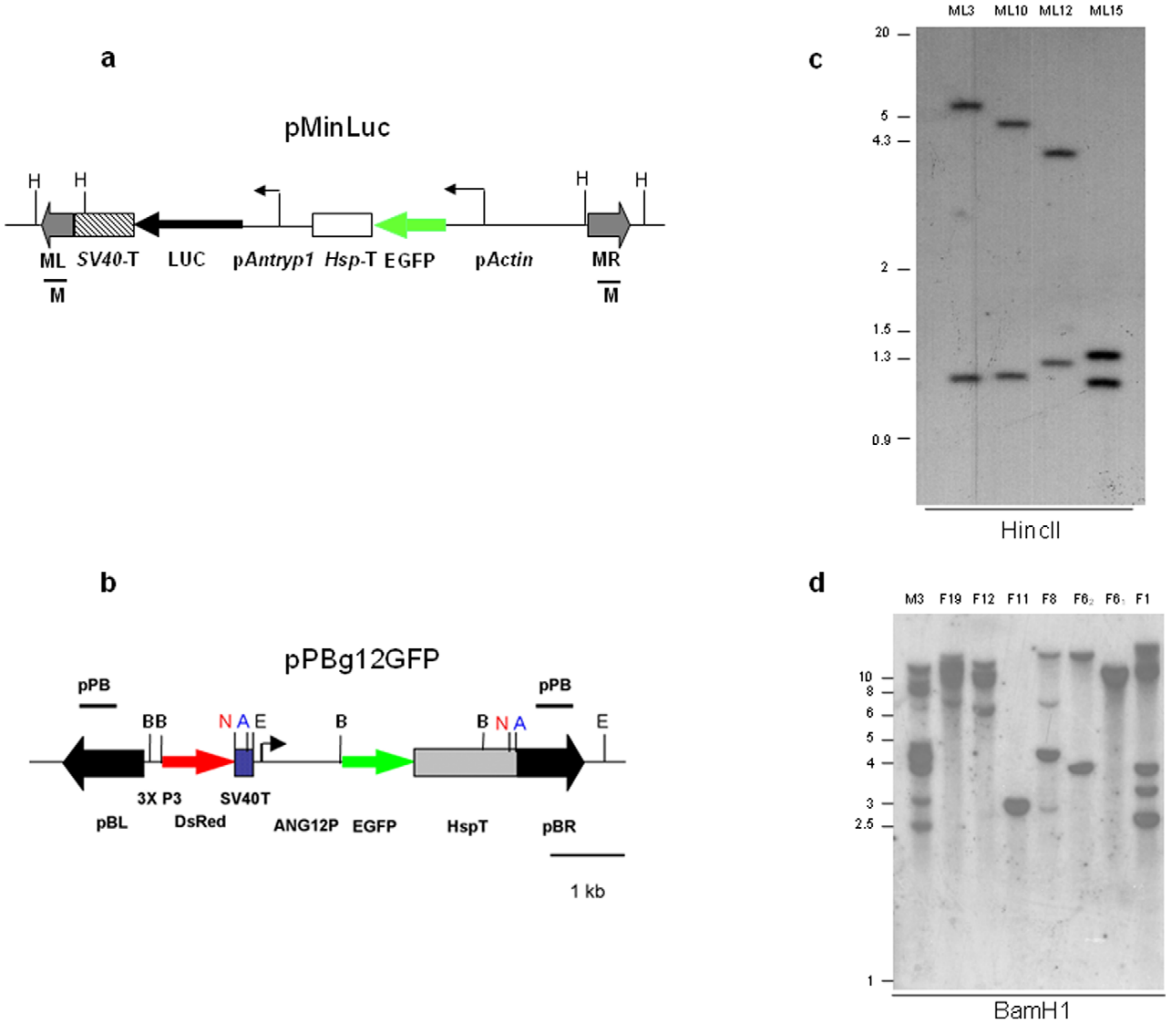
The *piggyBac* and *minos* transposable elements integrate into the genome of *A. stephensi*. Maps of transformation vectors pMinLuc (**a**) and pPBg12GFP (**b**). p*Actin*, *D. melanogaster Actin*5C promoter; *Hsp*T, *D. melanogaster Hsp*70 terminator sequence; p*Antryp1*, 1.1 kb of the upstream sequence of the *Antryp1* gene; *LUC*, luciferase gene; SV40T, SV40 polyadenylation signal; *3xP3*, artificial neuronal-specific promoter; dsRed, red fluorescent protein; ANG12P, 1.1 kb of putative *G12* promoter region pBL, *piggyBac* left arm; pBR, *piggyBac* right arm; ML, *minos* left arm; MR, *minos* right arm; H, *Hinc*II; E, *Eco*RI; B, *Bam*HI, N, *Not*I, A, *Asc*I. Black bars represent the probes (pPB and M) used in the Southern Blot analyses. **c**: Southern Blot analysis of *A. stephensi* lines AsML3, AsML10, AsML12 and AsML15 digested with *Hinc*II and hybridised with probe M (Figure 1a). Each individual transposon integration is expected to yield two hybridising bands. *Minos*-mediated lines were characterised by single transposon insertions **d**: Southern blot of *A.stephensi* lines transformed with pPBg12GFP. Genomic DNA from 8 different transgenic lines, resulting from 7 different founder families, was digested with *Bam*HI and hybridised with probe pPB, expected to give two hybridising bands per transposon insertion.

In order to assess the activity of the *Antryp1* promoter in *A. stephensi* mosquitoes, we generated a reporter transcription unit containing the luciferase gene (*luc*) placed under the control of the 1.1 kb region immediately upstream of *Antryp1* (pAntryp1). The selected promoter region spans nucleotides −1 to −1100 upstream of the start codon and encompasses most of the genomic region between the 5′ end of *Antryp1* and the 3′ end of Antryp2 lying adjacent in the trypsin cluster. The pAntryp1-luc fragment was cloned, together with an Actin5C-EGFP marker cassette, within the arms of the *minos* transposable element, to generate transformation vector pMinLuc [21](Figure 1a).

Transformation experiments yielded four separate founder females that produced transgenic lines, representing a transformation efficiency of 8.5% (Table S1). Segregation of the marker allele in each line indicated the occurrence of an insertion on a single chromosome (data not shown). Southern blot analysis of the 4 lines confirmed that a single integration of the transposable element had occurred in each line (Figure 1b). Two of the lines (AsML10 and AsML15) showed an insertion on the X chromosome, as assessed by both segregation data and *in situ* hybridisation analysis (data not shown). The *minos* integration site of transgenic line AsML10 was sequenced and the precise occurrence of *minos*-mediated integration confirmed (not shown, EMBL accession numbers AJ496323 and AJ496324).

**Figure 2:**
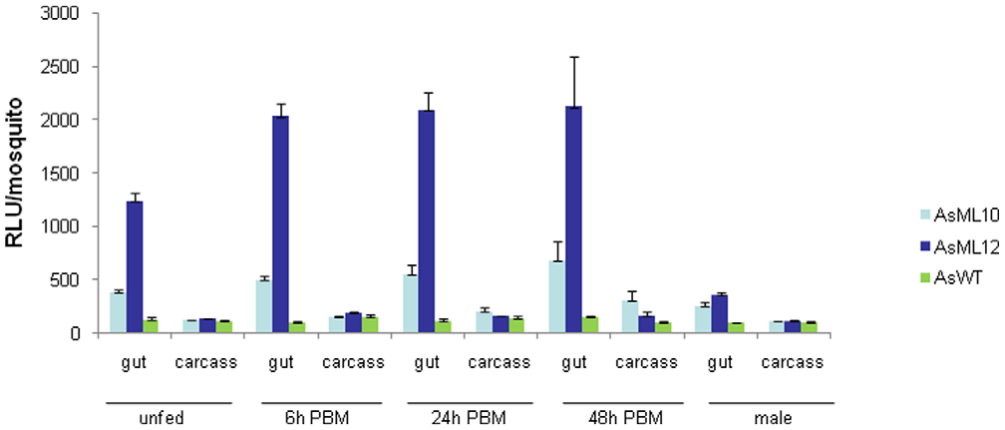
Blood meal inducibility and female predominance of transgenic luciferase activity driven by the *Antryp1* promoter. Comparison of luciferase activity in the transgenic lines AsML10 and AsML12 against wild type *A.stephensi* host (AsWT). Guts and carcasses from unfed females, females 6 h, 24 h, and 48 h post blood feeding and males were analysed individually. Bars show the mean of 20 samples for each condition. Error bars indicate the standard error of the mean.

To assess the tissue-specificity of the p*Antryp1* transcription unit, luciferase activity was analysed in detail in two *A. stephensi* transgenic lines, AsML10 and AsML12. Guts and carcasses of transgenic mosquitoes were dissected from males, unfed females and fed females at different time points after a blood meal, and assayed separately. Investigation of p*Antryp1* expression revealed luciferase activity in the gut of unfed females of both lines, with levels 3- and 9-fold higher than wild type controls, respectively (Figure 2). In both lines, levels of luciferase activity in the carcass were similar to control levels at all time points analysed, indicating that the *Antryp1* promoter is not active in those tissues. Luciferase activity was then assessed in female guts 6 hours after a blood meal. A 1.3- and 1.6-fold induction was observed in AsML10 and AsML12 respectively, when compared to unfed guts. At 24 h after a blood meal, when peak induction of endogenous *Antryp1* transcription is observed in *A. gambiae*, the levels of luciferase in the two *A. stephensi* lines were not significantly increased over those observed at 6 hours, and they remained constant at the 48 h time point. Preliminary analysis of p*Antryp1* activity in the two remaining *A. stephensi* lines, AsML3 and AsML15, showed luciferase levels similar to those detected in AsML10 (data not shown).

In all transgenic lines analysed, there was a significant level of luciferase activity in the guts from transgenic male individuals, although considerably lower than that detected in the unfed females (Figure 2). This observation is in agreement with previous reports documenting low but significant endogenous levels of *Antryp1* mRNA in the male guts of *A. gambiae* mosquitoes[17]. Despite the sex-bias, tissue-bias and bloodmeal induction of expression observed for the *pAntryp1-luc* cassette, other aspects of the endogenous *Antryp1* gene were poorly maintained. Endogenous *Antryp1* in *A. gambiae* is readily detectable by northern blot analysis upon blood feeding, yet we were unable to detect pAntryp1-driven luciferase transcripts in this fashion and the fold induction of luciferase expression was significantly less than that reported for the endogenous *Antryp1* [17]. Given that the low level of reporter gene expression was seen in all transgenic lines tested it is unlikely that it is due to positional effects related to the chromosomal location of the transgene. Together these observations suggest either that the *pAntryp1* region used in our construct, which contains most of the intervening region between *Antryp1* and *Antryp2*, lacks some control elements for efficient transcription (a frequent problem with promoters isolated from clusters where gene expression may be co-regulated) or that similarly to experiments with *Drosophila*, the *Antryp1* promoter from *A. gambiae* is not efficiently recognised in *A. stephensi*.

Interestingly, the endogenous late trypsin gene isolated from *A. stephensi* (*Astryp1*)[22] does not exhibit an identical expression profile to the homologous gene from *A. gambiae*. In particular, the blood-induction of *Astryp1* from *A. stephensi* peaks at 6 hours after a blood meal (Crisanti, unpublished data). Accordingly, the altered induction kinetics of p*Antryp1* observed after a blood meal in *A. stephensi* transgenic lines could reflect the normal activity of the transcriptional machinery in the two vector species. This is in agreement with what has been observed in transgenic *Aedes aegypti* mosquitoes, where the transcriptional profile of an *A. gambiae carboxypeptidase* (*AgCP*) promoter was shifted towards that of the endogenous *Ae. aegypti* gene [4]. Interestingly, in investigating the ability of the *pAntryp1* promoter to drive the *luc* reporter in its endogenous host, we managed to generate a single transgenic *A. gambiae* line that showed strong and bloodmeal-induced luciferase expression that was midgut-specific and peaked at 24 h post bloodmeal reminiscent of the endogenous *AgAntryp1* expression profile (Figure S1c). This observation lends weight to the hypothesis that some crucial *cis* and *trans* regulatory factors necessary for the correct functionality of pAntryp1 differ in *A. stephensi*. However we cannot rule out the possibility that the superior performance of this promoter construct in *A. gambiae* is due to position effects associated with the sequence context at the locus of transgene integration in this line.

### Validation of pG12 promoter activity in transgenic mosquitoes

To generate a pG12-reporter construct, 1.1 kb of the region immediately 5′ of the putative coding sequence of the *G12* gene was cloned upstream of the fluorescent reporter gene *EGFP*. This reporter construct was included in the *piggyBac* transformation vector pPB-G12EGFP, containing also the *3xP3-DsRed* marker gene (Figure 1c)[23]. Transformation experiments with pPBg12EGFP yielded 7 different founder families, representing a minimum transformation efficiency of ∼13% (Table S2). As has been noted in other reports of *piggyBac* transformations[24], [25], [26], [27], transformation events were characterised by multiple insertions (Figure 1d, which represents the transgenic lines at generation 5).

**Figure 3:**
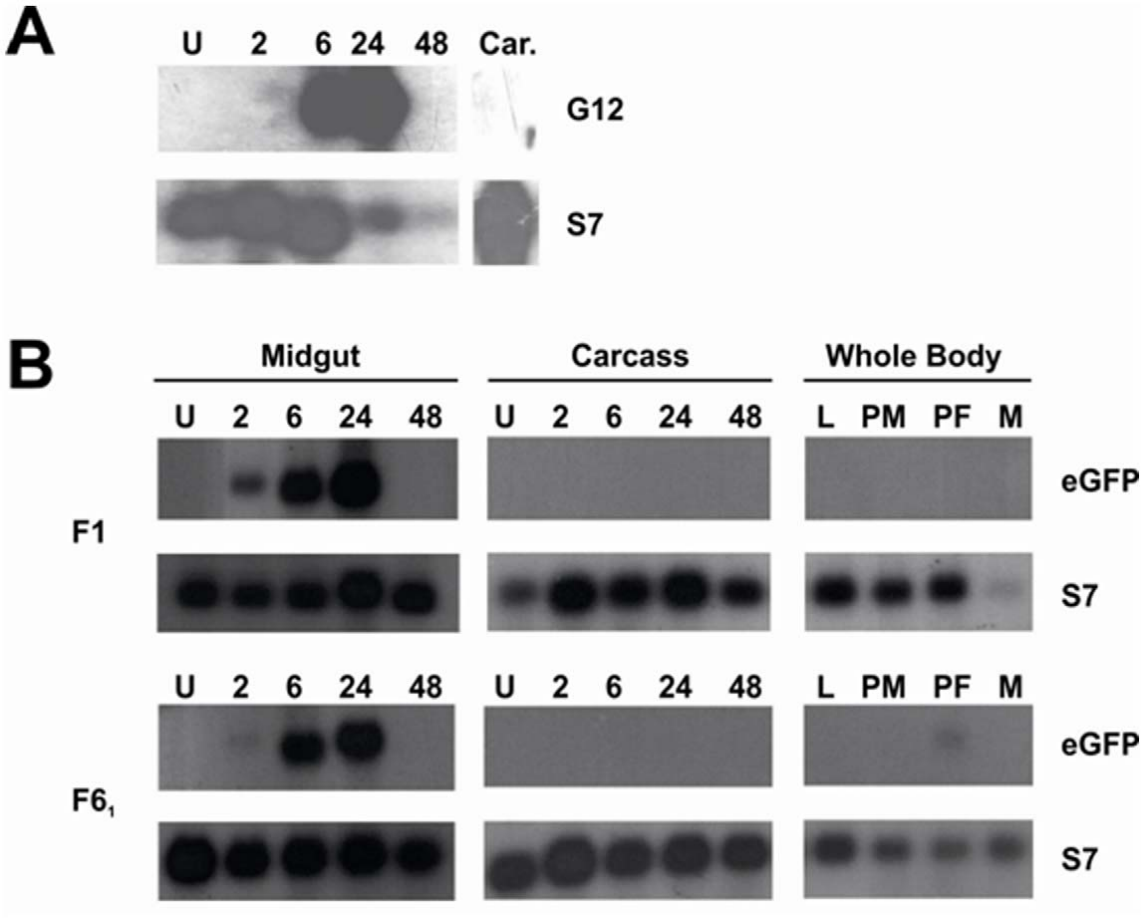
Reporter gene expression driven by the *G12* promoter closely resembles the transcriptional of the endogenous female-specific, bloodmeal-inducible G12 gene. (**a**) Northern blot analysis was performed to profile the expression of the endogenous *G12* gene in *A. gambiae*. We detected maximal expression in midguts 24 hours PBM. No expression was detected in the carcass at this timepoint (Car.), or at any other time point (not shown). As an internal loading control, blots were stripped and re-probed for the ubiquitous ribosomal *S7* gene. (**b**). Northern blot analysis of *EGFP* transcription under the control of the *A. gambiae G12* promoter in transgenic *A. stephensi.* Two independent transgenic lines (F1 and F6_1_) were tested. Stages and tissues tested were whole males (M), L3/L4 larvae (L), pupae (P), dissected midguts and carcasses unfed (U) and 2,6,24 and 48 hours PBM (2,6,24,48).

In order to fully characterise the expression pattern of the endogenous *G12* gene in *A. gambiae*, northern blot analysis was carried out at different developmental stages and at various time points after a bloodmeal. We detected a strong midgut-specific expression of *G12* in female adults, reaching peak levels of expression at 24 h PBM (Figure 3a). No expression was found in larvae, female carcasses or adult males, although a low level of expression was observed in the pupal stage (data not shown).

We thus examined reporter gene expression to see if the expression pattern of the endogenous *A. gambiae G12* gene was recapitulated in the transgenic *A. stephensi* lines transformed with pPBg12GFP containing the pG12-EGFP reporter cassette. We performed a detailed northern blot analysis of *EGFP* transcripts in two transgenic lines, one with a single transgene insertion (F6_1_) and one with multiple insertions (F1). In both cases expression was very similar to that of the endogenous *AgG12*, in terms of tissue- and sex-specificity and temporal profile in response to bloodmeal, with a clear peak at 24 h PBM (Figure 3b). These findings were confirmed in both lines by real time quantitative PCR experiments, which showed that reporter expression rose from being undetectable or less than 2% of the level of the abundant ribosomal *S7* control transcript to a peak at 100-fold greater than the same gene at 24 h PBM (Table S3).

**Figure 4:**
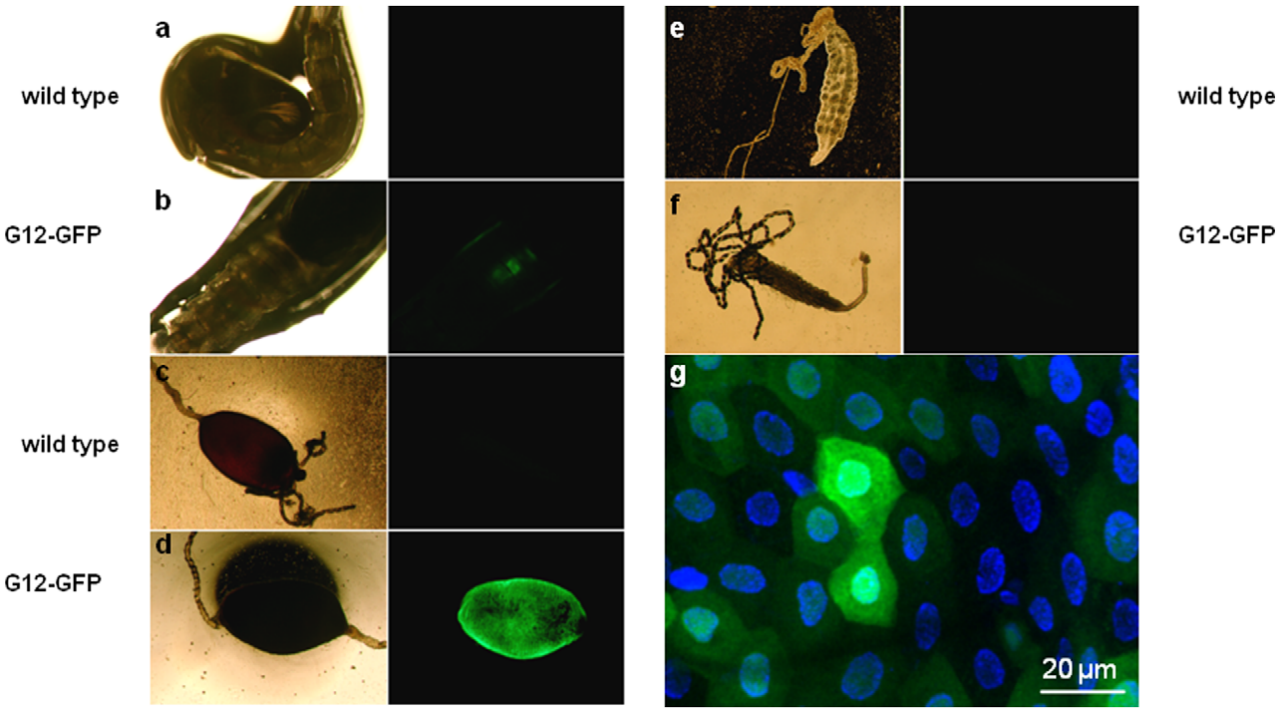
EGFP driven by the *G12* promoter accumulates in the midgut epithelium. Pupae (**a**,**b**), 24 h PBM midguts (**c**,**d**) and unfed female midguts (**e**,**f**) from wild type mosquitoes and a *pG12::EGFP* transgenic line (F6_1_) were visualised under transmission light (left panel) or epifluorescence (right panel). Reconstruction of a series of optical z-sections obtained by the Zeiss Apotome-assisted fluorescence microscopy (**g**) of 24 h PBM midguts dissected from transgenic mosquitoes reveals patchy distribution of EGFP-positive cells (green) across the epithelium. Cell nuclei are stained with DAPI (blue).

We then examined the tissue-specific accumulation of the EGFP protein expressed by the transgenic construct. Although the temporal pattern of EGFP accumulation will in part depend on the maturation kinetics and stability of the protein, the visualisation of this protein allows a full evaluation at the microscopic level of the tissue-specific localisation afforded by the G12 promoter. The utility of this promoter for directing tissue-specific transgene expression is supported by the fact that each of the 8 separate transgenic lines containing the construct showed a similar pattern of accumulation of the EGFP protein in the midgut in response to bloodmeal. This was clearly visible in midguts dissected at 24 h PBM while no signal was invisible in the unfed state or in any other tissue (see Figure 4a–f). Moreover, EGFP expression was also visible at the pupal stage, mirroring the endogenous G12 transcriptional profile. No expression was detected in the male (not shown). Although the pattern of EGFP accumulation was remarkably consistent, the intensity of the signal showed some variation between lines, presumably due to position effects associated with transgene insertion and/or to differences in number of *piggyBac* insertions.

To examine pG12-driven accumulation of EGFP at the cellular level in the midgut epithelia we used fluorescence microscopy at high magnification. The mosquito midgut epithelium is composed of a single layer of cells. Interestingly, the distribution of GFP-expressing cells was non-uniform, with some cells strongly fluorescent surrounded by others showing intermediate or no EGFP expression (Figure 4g). This patchy expression profile suggests that while the high levels of inducible expression afforded by the *G12* promoter bode well for the expression of secreted effector proteins active against stages of the *Plasmodium* parasite present within the midgut lumen, this promoter may not be suitable for the expression of molecules designed to be active intracelullarly against ookinetes as they traverse the midgut epithelium. This observation highlights the importance of microscopic analysis at the level of individual cells in determining the suitability of tissue-specific reporters for their purpose. Given this finding, it will be interesting to determine at the cellular level the expression of previously reported midgut-specific promoters used to drive expression of transgenes[2], [12], [14].

Since many anti-*Plasmodium* effector molecules are likely to have some deleterious effects on the mosquito as well as the parasite, it is desirable as a strategy to minimise expression of the effector to those tissues that encounter the parasite. In this light the bloodmeal inducibility and strict female midgut-specificity imparted by the pG12 construct give it a potential advantage over constructs containing promoter regions from other bloodmeal-inducible *A. gambiae* genes such as *AgCP* and *Aper1*, which show some constitutive activity in the male gut or the female gut or both[2], or the *Antryp1* studied here.

Interestingly, there is evidence that expression of *G12* in *Anopheles gambiae* and *Aedes aegypti* is massively induced in the midgut in response to a bloodmeal,yet the fold of this induction can be conditioned to some extent both by the presence of *Plasmodium* in a bloodmeal and by the refractory status of the mosquito[20], [28]. Further studies will assess whether this issue needs to be considered when using pG12 to drive expression of anti-*Plasmodium* effector molecules. pG12-reporter constructs may therefore be a valuable tool in studying any modulatory effect of the insect immune response on *G12* expression.

### Conclusions

We have described two promoter regions from *A. gambiae* that are able to direct, with different characteristics, bloodmeal-induced, midgut-enriched transgene expression in *A. stephensi*. In particular, pG12 is anticipated to be of great use in *Anopheles* species for tailoring transgene expression to coincide with midgut stages of the *Plasmodium* parasite, for the purposes of functional studies on mosquito-parasite interaction and for novel malaria control approaches through the targeted expression of anti-*Plasmodium* effector molecules.

## Methods

### Ethics statement

All animal work was conducted according to UK Home Office Regulations and approved under Home Office License PPL 70/6453.

### Plasmid construction

Plasmid pMinLuc (Figure 1a) was derived from plasmid pMinEGFP[29] by inserting a *Not*1 cassette containing 1.1 kb of the region immediately upstream of the start codon of *Antryp1* (AGAP008296)[22], amplified from genomic DNA from *A.gambiae* Suakoko strain, followed by the 1.7 kb *Photinus pyralis* luciferase cDNA[30] and SV40 polyadenylation signal.

The piggyBac-based plasmid pPBLuc (Figure S1a), containing identical reporter gene and marker elements to MinLuc used to transform *A. gambiae* was created by sequentially cloning the p*Antryp1*-luciferase and the *Actin*5C-EGFP cassettes from pMinLuc as *Not*1 fragments into the *piggyBac*-based plasmid pPBKOα*Not*I^24^

Plasmid pPBg12GFP was created by amplifying a 1.1 kb of the region immediately upstream of the *G12* gene (AGAP006187) from the *A. gambiae* KWA strain (London School of Tropical Hygiene and Medicine) using the primers pG12forward (5′-ccc*gaattc*cacaataccggccctgaa-3′) and pG12reverse (5′-ggg*ggatcc*gatgctgatgattggattgg-3′), designed to include *Eco*R1 and *Bam*H1 restriction sites, respectively (*italics*). The amplified product was ligated as a *Eco*R1/*Bam*H1 fragment to the *EGFP* gene in pMinEGFP[29] and a fragment containing the pG12, *EGFP* and the SV40 terminator sequence was inserted into the final transformation vector pBac[3xP3-DsRedaf][23], which includes the transformation marker DsRed driven by the neuronal-specific synthetic promoter 3xP3.

The helper plasmids pHSS6hsILMi20 and phsp-pBac, respectively providing the *Minos* and the *piggyBac* transposase genes, have previously been described[31], [32].

### Embryo microinjection

To complete their gonotrophic cycle mosquitoes were fed on anaesthetized mice, in accordance with UK Home Office guidelines. *A. stephensi* embryos (strain sd500) were injected with a mixture of helper plasmid pHSS6hsILMi20 (100 µg/ml) and transformation vector pMinLuc (400 µg/ml) or a mixture of helper plasmid phsp-pBac (100 µg/ml) and pPBg12GFP (400 µg/ml), as previously described[29]. *A. gambiae* embryos (KWA strain, London School of Hygiene and Tropical Medicine) with a mixture of helper plasmid phsp-pBac (100 µg/ml) and pPBLuc (400 µg/ml). Hatched larvae were analysed on an inverted microscope under epifluorescence using either a GFP filter or a Texas Red filter to detect dsRED expression, depending on the transformation experiment.

### Southern blot analyses

Approximately 4 µg of genomic DNA from transgenic *A. stephensi* adults was digested with the restriction endonuclease *Hinc*II (MinLuc lines), *Bam*HI (PBg12GFP lines) or *Eco*R1(AgPBLuc line)and blotted according to standard protocols. Digested genomic DNA was separated on a 0.8% agarose gel and transferred onto a nylon membrane. The membranes were hybridised overnight at 65°C with different ^32^P-labelled probes. In the case of the *minos*-mediated *A. stephensi* transformations, genomic DNA was hybridised with the previously described probe M (Figure 1a)[29], encompassing both the left and the right arms of *minos*. In the case of the *piggyBac*-mediated PBg12GFP transformations, and the A.gambiae PBLuc line, the previously described probe PB[26], encompassing sequences of the left and right arms of the *piggyBac* transposon, was utilised (Figure 1c and Figure S1b).

### Northern blot analysis

10 µg of total RNA was separated by electrophoresis on a 0.8% denaturing formaldehyde agarose gel and blotted to HYBOND™ nylon membrane (Amersham) through capillary transfer according to manufacturers protocol. Blots were hybridised overnight at 42°C in formamide-based hybridisation buffer with a ^32^P labelled probe corresponding to the entire *G12* or *EGFP* coding sequence to probe for the endogenous *G12* transcripts or the pG12-driven transgenic transcripts, respectively. As a loading control a similar probe was prepared corresponding to the coding sequence of the ribosomal gene *S7*.

### Microscopy

Mosquito tissues were dissected in PBS and EGFP expression was visualised under epifluorescence using a EGFP filter.

Fluorescence microscopy of the midgut epithelium was performed on midguts dissected 24 h PBM in PBS and rinsed to remove blood bolus. Midguts were fixed in methanol-free 4% formaldehyde (Pierce) in PBS for 30 min at room temperature and washed 3 times for 15 min in 0.1% Tween-20 PBS. Midguts were then mounted on slides containing Vectashield mounting medium with DAPI (Vectorlabs. Inc.) with cover slips. Multiplane z-stacks were collected by a Zeiss fluorescence microscope Axiovert 200 M equipped with a Zeiss Apotome module. (www.Zeiss.com).

### Luciferase Assay

Four- to five-day old mosquitoes were dissected to separate the midgut from the carcass. Each sample was homogenised in 150 µl of Cell Culture Lysis Reagent (Promega), supplemented with 5 mM phenylmethylsulfonylfluoride and 7% reconstituted milk powder to block degradation of luciferase by endogenous mosquito protease activity. Immediate assay of the samples in the presence of reconstituted milk powder was essential for luciferase detection in female guts. A volume of 30 µl of homogenate was added to 100 µl of Luciferase Assay Substrate (Promega), and light emission measured for 15 seconds with a Berthold luminometer. Twenty guts and carcasses were assayed for each time point.

## Supporting Information

Figure S1 **Generation and analysis of transgenic** ***A.gambiae*** **line AgPB1.** **A:** map of transformation vector pPBLuc. p*Actin*, *D. melanogaster Actin*5C promoter; *Hsp*T, *D. melanogaster Hsp*70 terminator sequence; p*Antryp1*, 1.1 kb of the upstream sequence of the *Antryp1* gene; LUC, luciferase gene; SV40T, SV40 polyadenylation signal; pBL, *piggyBac* left arm; pBR, *piggyBac* right arm; E, *Eco*RI. Black bars represent the probe (PB) used in the Southern Blot analyses. **B:** Southern Blot analysis of genomic DNA from the *A. gambiae* line AgPB1, digested with *Eco*RI and hybridised with probe P (Figure 1A). Each single insertion is expected to give two hybridising bands, corresponding to each arm of the piggyBac element. **C:** Comparison of luciferase activity in *A. gambiae* line AgPB1 with the transgenic *A. stephensi* lines AsML10 and AsML12. Guts and carcasses from unfed females (U), females 6 h, 24 h, and 48 h post blood feeding and males were analysed individually. Bars show the mean of 20 samples for each condition. Error bars indicate the standard error of the mean.
(TIF)

Table S1 **Generation of transformants using plasmid pMinLuc.** The 47 surviving adults from a total of 167 injected embryos were outcrossed with wild type *A. stephensi* in groups of same-sex individuals. The 24 females from the female group were allowed to lay eggs in isolation to determine the number of single founders. 4 female founders produced fluorescent individuals among their G1 progeny, indicative of a germline integration event. Transgenic progeny were interbred to achieve homozygosity of the transgene. Asterisks denote those lines that were assayed in detail for luciferase activity.
(DOCX)

Table S2 **Generation of transformants using pPB-G12EGFP.** The 55 surviving adults from a total of 391 injected embryos were outcrossed with wild type *A. stephensi* in 4 groups of same-sex individuals. The 31 females from the female group were allowed to lay eggs in isolation (F1-F31) to determine the number of single founders. Segregation patterns of the transgene in subsequent outcrossing experiments revealed that many lines contained multiple transgene insertions at separate loci. Where possible lines were bred to achieve homozygosity at the transgenic loci.
(DOCX)

Table S3 **Quantitative real time PCR analysis of EGFP reporter expression driven by the G12 promoter in** ***A. stephensi*** **.** Quantification was performed using TaqManTM primers and probes (ABI) specific for the EGFP gene and the S7 ribosomal gene as a reference control, as previously described [1]. The PCR cycle number (Ct) at which a threshold of amplification was calculated for each gene and the level of EGFP was calculated relative to the S7 internal control using the delta Ct method [2]. **Supplementary References** 1. Brown, A. E., Bugeon, L., Crisanti, A. & Catteruccia, F. Stable and heritable gene silencing in the malaria vector Anopheles stephensi. Nucleic Acids Res 31, e85 (2003). 2. Schmittgen, T. D. & Livak, K. J. Analyzing real-time PCR data by the comparative C(T) method. Nat Protoc 3, 1101-8 (2008).
(DOCX)
