# Supplementary figures and images for: Analysis of Two Novel Midgut-Specific Promoters Driving Transgene Expression in Anopheles stephensi Mosquitoes
Source: PLoS One. 2011 Feb 4;6(2):e16471. doi: 10.1371/journal.pone.0016471 (PMC3033896; doi:10.1371/journal.pone.0016471)

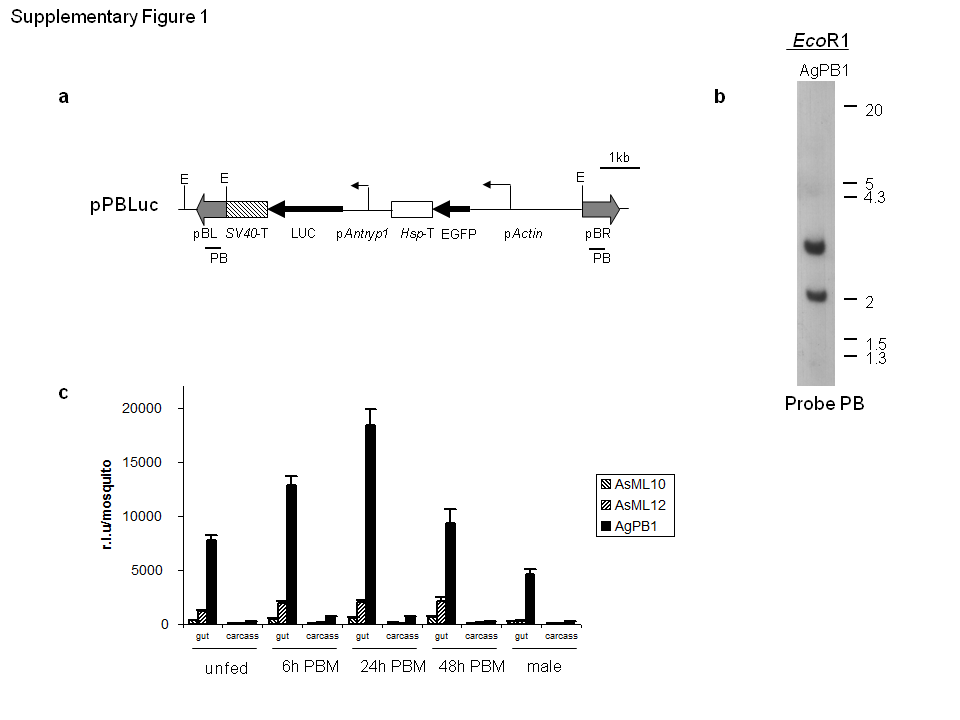

Supplement: Figure S1 — Generation and analysis of transgenic A.gambiae line AgPB1. A: map of transformation vector pPBLuc. pActin, D. melanogaster Actin5C promoter; HspT, D. melanogaster Hsp70 terminator sequence; pAntryp1, 1.1 kb of the upstream sequence of the Antryp1 gene; LUC, luciferase gene; SV40T, SV40 polyadenylation signal; pBL, piggyBac left arm; pBR, piggyBac right arm; E, EcoRI. Black bars represent the probe (PB) used in the Southern Blot analyses. B: Southern Blot analysis of genomic DNA from the A. gambiae line AgPB1, digested with EcoRI and hybridised with probe P (Figure 1A). Each single insertion is expected to give two hybridising bands, corresponding to each arm of the piggyBac element. C: Comparison of luciferase activity in A. gambiae line AgPB1 with the transgenic A. stephensi lines AsML10 and AsML12. Guts and carcasses from unfed females (U), females 6 h, 24 h, and 48 h post blood feeding and males were analysed individually. Bars show the mean of 20 samples for each condition. Error bars indicate the standard error of the mean. (TIF) [file pone.0016471.s001.tif]
