# Supplementary material for: Analysis of Two Novel Midgut-Specific Promoters Driving Transgene Expression in Anopheles stephensi Mosquitoes
Source: PLoS One. 2011 Feb 4;6(2):e16471. doi: 10.1371/journal.pone.0016471 (PMC3033896; doi:10.1371/journal.pone.0016471)
Supplement: Table S1 — Generation of transformants using plasmid pMinLuc. The 47 surviving adults from a total of 167 injected embryos were outcrossed with wild type A. stephensi in groups of same-sex individuals. The 24 females from the female group were allowed to lay eggs in isolation to determine the number of single founders. 4 female founders produced fluorescent individuals among their G1 progeny, indicative of a germline integration event. Transgenic progeny were interbred to achieve homozygosity of the transgene. Asterisks denote those lines that were assayed in detail for luciferase activity. (DOCX) [file pone.0016471.s002.docx]

## Table S1. Generation of transformants using plasmid pMinLuc.

| Adults | wt outcross | Group/ founder | Fluorescent/total G_1_s |
| --- | --- | --- | --- |
| 47 | 23 males  24 females | -  A  AsML3  AsML10^**^  AsML12^*^  AsML15 | 0/1199  0/1380  41/61  18/152  45/148  12/296 |
