# Supplementary material for: Analysis of Two Novel Midgut-Specific Promoters Driving Transgene Expression in Anopheles stephensi Mosquitoes
Source: PLoS One. 2011 Feb 4;6(2):e16471. doi: 10.1371/journal.pone.0016471 (PMC3033896; doi:10.1371/journal.pone.0016471)
Supplement: Table S2 — Generation of transformants using pPB-G12EGFP. The 55 surviving adults from a total of 391 injected embryos were outcrossed with wild type A. stephensi in 4 groups of same-sex individuals. The 31 females from the female group were allowed to lay eggs in isolation (F1-F31) to determine the number of single founders. Segregation patterns of the transgene in subsequent outcrossing experiments revealed that many lines contained multiple transgene insertions at separate loci. Where possible lines were bred to achieve homozygosity at the transgenic loci. (DOCX) [file pone.0016471.s003.docx]

# Table S2 Generation of transformants using pPB-G12EGFP.
