# Supplementary material for: Analysis of Two Novel Midgut-Specific Promoters Driving Transgene Expression in Anopheles stephensi Mosquitoes
Source: PLoS One. 2011 Feb 4;6(2):e16471. doi: 10.1371/journal.pone.0016471 (PMC3033896; doi:10.1371/journal.pone.0016471)
Supplement: Table S3 — Quantitative real time PCR analysis of EGFP reporter expression driven by the G12 promoter in A. stephensi . Quantification was performed using TaqManTM primers and probes (ABI) specific for the EGFP gene and the S7 ribosomal gene as a reference control, as previously described [1]. The PCR cycle number (Ct) at which a threshold of amplification was calculated for each gene and the level of EGFP was calculated relative to the S7 internal control using the delta Ct method [2]. Supplementary References 1. Brown, A. E., Bugeon, L., Crisanti, A. & Catteruccia, F. Stable and heritable gene silencing in the malaria vector Anopheles stephensi. Nucleic Acids Res 31, e85 (2003). 2. Schmittgen, T. D. & Livak, K. J. Analyzing real-time PCR data by the comparative C(T) method. Nat Protoc 3, 1101-8 (2008). (DOCX) [file pone.0016471.s004.docx]

**Table S3 Quantitative real time PCR analysis of EGFP reporter expression driven by the G12 promoter in *A. stephensi***

**F1**

**F6_1_**
